# Children’s Health: Soil in the City: A Prime Source of Lead

**DOI:** 10.1289/ehp.116-a522a

**Published:** 2008-12

**Authors:** Lance Frazer

Leaded gas was phased out in 1986, lead-based house paint a decade earlier. As a result, the percentage of U.S. children aged 5 years and younger exceeding the 10-μg/dL blood lead level of concern established by the Centers for Disease Control and Prevention has dropped dramatically. On a national average, the rate fell from over 80% in 1976 to just below 2% in 2002. In many cities, however, it still commonly exceeds 15%. Why? A review published in the August 2008 issue of *Applied Geochemistry* points to urban soil as a lingering source of lead poisoning in children.

Review coauthor Gabriel Filippelli, chair of the Earth Sciences Department at Indiana University–Purdue University, Indianapolis, believes elevated blood lead levels in inner-city children come largely from exposure to lead dust that has settled over decades, resulting in soil whose lead content, at the most contaminated spots, can reach 100 times the background level of about 50 ppm. Children are typically exposed to this lead through play and hand-to-mouth activity.

In research published in the 15 December 2007 *Science of the Total Environment* (which was not included in the review article), Tulane University research professor and environmental toxicologist Howard W. Mielke and colleagues revealed a curvilinear relationship between soil lead in New Orleans and children’s blood lead. The rate of increase was around 3 μg/dL for every 1,000 ppm soil lead above 250–400 ppm, but the rate of increase of blood lead was 4 times higher at soil lead concentrations below 100 ppm. High lead levels damage children’s nervous systems, stunt growth, delay development, and contribute to later-life delinquent behavior.

Filippelli mapped soil lead levels near Indianapolis roadways, tracking changes from older inner-city areas toward the newer suburbs. “In places such as the newer neighborhoods, far away from the urban core, where roads weren’t so heavily traveled before leaded gas was eliminated, you have noticeably lower lead levels,” he says. “And when we overlaid a 1990s study of lead poisoning in children to the map we had developed, the distribution of cases fit our pattern precisely.” This study, published with review coauthor Mark A.S. Laidlaw in the June 2005 issue of *EHP*, also showed that inner-city children had higher blood lead levels in the drier late summer months and lower levels in the winter. “We developed a meteorological prediction model that not only fit the results of our study over ninety percent of the time, but also showed a high degree of correlation with other cities as well,” says Filippelli.

The review also looked at options for remediation of lead-contaminated urban soil. Removal of lead-contaminated soils and replacement with clean topsoil can cost more than $1 million per acre, says Filippelli, who proposes two alternatives. “The simplest and least expensive would be a program of high-power shower-spraying of clean water during the months when soil conditions are at their driest, to keep lead-tainted soils from blowing onto horizontal surfaces and into homes.” A second approach would be to put in a layer of clean soil and then hydroseed with grass. Filippelli plans additional experiments in Indianapolis next summer to gain a better picture of the cost and effectiveness of these and other options.

“I suspect that Filippelli’s second proposal will be more effective and longer-lasting and—given the water supply issues of many cities—probably more attractive,” says Mielke. In a study reported 15 April 2006 in *Environmental Science & Technology*, Mielke and colleagues reported success in covering heavily contaminated soils in New Orleans with low-lead alluvium from the Mississippi River.

The cost of remediation is certainly a factor, but the cost of inaction is also high. “Aggregate public health costs from lead poisoning in New Orleans alone run more than $70 million annually,” says Mielke, who points to proactive steps being undertaken in Norway. “The Norwegian government has begun testing and remediating soils at day care centers, elementary schools, and parks in major cities, while we dance around issues of responsibility and cost, spending millions in health care costs after using our children as bioindicators.”

